# The Influence of Social Distancing Behaviors and Psychosocial Factors on Physical Activity During the COVID-19 Pandemic: Cross-sectional Survey Study

**DOI:** 10.2196/31278

**Published:** 2021-09-24

**Authors:** Troy J Cross, Jennifer M J Isautier, Sarah J Morris, Bruce D Johnson, Courtney M Wheatley-Guy, Bryan J Taylor

**Affiliations:** 1 Sydney School of Health Sciences Faculty of Medicine and Health The University of Sydney Camperdown Australia; 2 Department of Cardiovascular Diseases Mayo Clinic Rochester, MN United States; 3 Department of Cardiovascular Diseases Mayo Clinic Scottsdale, AZ United States; 4 Department of Cardiovascular Diseases Mayo Clinic Jacksonville, FL United States

**Keywords:** physical activity, COVID-19, mental health, social distancing, public health, pandemic, physical health, exercise

## Abstract

**Background:**

The COVID-19 pandemic has arguably facilitated a shift toward increased sedentariness and reduced physical activity. Moreover, there is mounting evidence that mental health has also declined during the pandemic. However, it remains unknown to what extent social distancing (SD) behaviors and mental health have affected the physical activity levels of the general population.

**Objective:**

The purpose of this study was to determine the influence of SD behaviors and prevailing mental health on the odds of being physically active during the early COVID-19 pandemic response.

**Methods:**

A total of 4819 adults (2474/4819, 51.3%, female) from the US population with a median age of 46 (IQR 35-59) completed an online survey during the early pandemic response (April-June 2020). The survey included questions on adherence to 11 SD behaviors, and validated questionnaires which assessed self-reported physical activity, depression, anxiety, and mental well-being. Respondents were categorized into 2 physical activity groups: inactive (0-599 metabolic equivalent of task [MET]-minutes/week) and active (≥600 MET-minutes/week). A logistic generalized additive model (GAM) was used to determine which SD factors and mental health outcomes were associated with physical activity level.

**Results:**

The GAM analysis revealed that wearing a facemask in public (odds ratio [OR] 1.46, 95% CI 1.14-1.79; *P*=.003), limiting the use of public transport (OR 1.47, 95% CI 1.19-1.83; *P*=.001), and restricting travel outside the house (OR 1.56, 95% CI 1.19-2.05; *P*=.002) were SD behaviors associated with higher odds of being more physically active. Conversely, avoiding physical activity outside the house was associated with higher odds of being inactive (OR 0.52, 95% CI 0.46-0.63; *P*<.001). Leaving the house more frequently, and a higher mental well-being were associated with increasing odds of being physically active (*P*<.001). Engaging with a moderate number of SD behaviors (3-7 total) was positively associated with physical activity, whereas a very high SD vigilance (ie, engaging with ≥10 total behaviors) decreased the odds of being active during the early pandemic response.

**Conclusions:**

Based on the findings of our study, we suggest that future public health messaging of SD guidelines should include (1) a clear portrayal of the benefits of regular exercise on mental health; and (2) a specific focus on how to be physically active outdoors in a COVID-safe manner.

## Introduction

The COVID-19 outbreak was officially declared a pandemic on March 11, 2020, by the World Health Organization (WHO). During the first year of the COVID-19 pandemic response, no effective pharmaceutical therapies existed to prevent or contain the spread of the novel coronavirus. Consequently, many countries around the world began to rapidly implement nonpharmaceutical interventions to mitigate community transmission of COVID-19. These public health interventions included rules or guidelines for personal hygiene, respiratory etiquette, and social distancing (SD) [1-4]. On this latter point, SD is a broad term that encompasses many social behaviors designed to minimize interpersonal contact within the community, including but not limited to self-quarantine, working from home, school closures, restrictions on mass gatherings and travel outside the home, and minimum separation distance between persons in public spaces. In some circumstances, these public health measures have led governing authorities to enforce closure of local gymnasiums, sporting and recreational facilities, in addition to suspending organized team sports and other physical activities that would otherwise incur close interpersonal contact (dance classes, yoga, etc).

It follows from the above that SD guidelines and restrictions have reduced the opportunities for the public to engage in physical activity during the early COVID-19 pandemic. There is mounting evidence to suggest that physical activity has decreased since the beginning of the COVID-19 outbreak [5-10]. This shift toward sedentariness is especially alarming, seeing that sedentariness and physical inactivity are both well-known risk factors for long-term outcomes such as cardiovascular disease and premature mortality [11,12]. Physical inactivity had already been identified as a global pandemic itself prior to the COVID-19 outbreak [13]. Physical inactivity is the fourth leading risk factor for global mortality [14], and is perhaps of greater importance for the older rather than younger population during the current pandemic [15]. While SD is a necessary measure to minimize community transmission of COVID-19, it is important to also understand its collateral adverse effects such as reduced engagement in physical activity. In so doing, we may identify key areas for improving the messaging of SD guidelines in a way that ensures public safety, yet facilitates and encourages a healthy, active lifestyle as the pandemic continues.

It is noteworthy that opportunities for socialization through physical activity (eg, gym classes, team sports) have decreased during the COVID-19 pandemic. Moreover, SD measures are, by nature, a collection of behaviors specifically designed to minimize interpersonal contact, further diminishing opportunities for social interaction. Certainly, these fewer interactions may contribute to growing feelings of social isolation and loneliness during the pandemic [16]. The prolonged experience of social isolation may precipitate a poor state of mental health [17] which, in turn, may explain the increased symptoms of depression, anxiety, or reduced mental well-being reported during the pandemic [18-21]. It must be remembered that physical activity and mental health are related via a bidirectionally causal relationship [22,23]. As such, it is important that any investigation into the effects of SD behavior on physical activity during the COVID-19 pandemic is interpreted with consideration of the mental health status of the population under study.

We conducted a large, online cohort study among US residents. The principal aim of this study was to examine whether SD behaviors were associated with physical activity participation during the early phase of the COVID-19 pandemic (April to June 2020). A secondary aim was to examine the independent effects of mental health status on physical activity. We hypothesized that engaging in more SD behaviors, and having poorer mental health, would be associated with lower odds of meeting the minimum WHO recommendations for physical activity during the early COVID-19 pandemic response.

## Methods

### Study Design, Sampling, and Participant Recruitment

The data used in this study were drawn from a larger, longitudinal cohort study that commenced in April 2020: the COVID-19 Physical Activity and Well-being Survey (PAWS). The primary aim of the broader PAWS project is to examine temporal trends in physical activity and mental health throughout the COVID-19 pandemic in the United States. Data for this study were obtained from the first round of the PAWS. Participants were invited to complete the first round of the PAWS between April 27 and June 8, 2020. Survey responses were collected via the Qualtrics online platform. Participants were recruited via word of mouth, and social media campaigns (Facebook and Twitter) that were targeted using paid advertisements to recruit men and women across a wide range of ages. Participants were eligible to participate if they were aged 18 years or older, could read and understand English, and were able to provide a valid zip code as evidence of residing in the United States.

### Ethical Approval and Informed Consent

This study was approved by the Mayo Clinic Institutional Review Board (#20-003709). Participants were provided with an information sheet on the landing page of the online survey. Participants were only allowed to continue participating if they acknowledged that they had read the information sheet, and agreed to the following statement “I give consent to participate in this study”.

### Definition of Variables

#### Outcome

The outcome variable in this study was self-reported physical activity. Moderate-to-vigorous physical activity (MVPA) was determined using the Global Physical Activity Questionnaire (GPAQ) [24]. The GPAQ assesses the weekly volume of MVPA (minutes/week) in the domains of work, recreation, and transport. Data obtained from the GPAQ were cleaned and subsequently analyzed using the guidelines outlined by the WHO [25]. Weekly MVPA was expressed as an intensity-weighted volume (ie, metabolic equivalent of task [MET]-minutes/week) by multiplying moderate and vigorous activities by the corresponding metabolic equivalents of tasks (METs) of 4.0 and 8.0, respectively [24,25]. Total weekly MVPA (MET-minutes/week) was taken as the sum of all domain-specific MVPA, and was used to categorize participants into the following 2 groups: inactive (0-599 MET-minutes/week) and active (≥600 MET-minutes/week). These demarcations are based on the lower threshold of the minimum requirement outlined in the 2020 WHO physical activity guidelines [26].

#### Exposures

The 2 exposures of this study were linked to our primary and secondary aims: SD behaviors and mental health.

#### Social Distancing

SD behavior was assessed by asking participants to indicate if they were presently engaging in one (or any) SD behavior at the time of the survey (11 different behaviors in total; Textbox 1). The total number of SD behaviors (sum of SD1–SD11) was taken as an index of SD vigilance.

**Questions designed to assess participant engagement with social distancing behaviors at the time of the survey.**

**Social distancing behaviors**

“Regardless of whether specific guidelines/rules for social distancing have been issued by authorities in the place where you live, please indicate whether you are currently performing any of the following behaviors listed below. Please check all behaviors that apply.”

SD1: Wearing a face mask in public

SD2: Avoiding close contact with others in your social circle

SD3: Avoiding places where many people gather

SD4: Working from home

SD5: Limiting time spend outside of your residence

SD6: Limiting your use of public transport

SD7: Self-quarantine/isolation

SD8: Restricting your travel outside the house

SD9: Avoiding physical activity outside the house

SD10: Avoiding physical contact with others (ie, handshaking, hugging)

SD11: Reducing the time or number of trips to shop for food/supplies/etc

#### Mental Health

Mental health was evaluated by assessing participant’s symptoms of depression, anxiety, and mental well-being using the following tools: the 9-item Patient Health Questionnaire (PHQ-9) [27], the 7-item Generalized Anxiety Disorder scale (GAD-7) [28], and the 5-item World Health Organization Well-Being (WHO-5) Index [29,30], respectively.

### Covariates

#### Pandemic Burden and Fear

We assessed the zip code–level burden of the COVID-19 pandemic by obtaining the number of deaths, and the confirmed and recovered cases attributed to the disease. These data were obtained from an up-to-date, online repository of COVID-19–related information [31]. Using this repository, we were able to obtain case numbers for each respondent’s US state using zip code provided by the participant and the date the survey was completed. The difference between confirmed and recovered COVID-19 cases was taken as the number of active cases in the area. Deaths and case numbers were expressed per capita of the state in which the participant resided. Furthermore, we calculated the duration of time that SD guidelines/restrictions had been imposed by taking the difference between the survey response date and the first date in which the “stringency index” [32] of the participant’s state was greater than 0. The “stringency index” is a novel score indicating the stringency with which a local government is responding to the COVID-19 pandemic. It is computed from a weighted average of 9 metrics used to characterize the strictness of containment and closure policies of the area. Participants were also asked to indicate their current level of fear associated with being infected by, or unknowingly spreading COVID-19.

#### Sedentary and Self-Monitoring Behavior

Data on sedentary behavior (minutes/day) were obtained directly from the GPAQ [24,25]. These data were used to categorize participants into 2 groups defined around an approximate threshold associated with increased cardiovascular morbidity (≥8 hours/day) in harmonized pooled studies [33]. Participants were asked to indicate whether they currently used a wearable device to track their physical activity.

#### Socioeconomic Status and Physical Health

Socioeconomic status [34,35], physical health [36-38], and chronic disease [39,40] are known to influence physical activity. Accordingly, sociodemographic variables, including age, gender, height, weight, educational, and employment status were collected, in addition to self-reported chronic disease and overall health status. Breathlessness, a hallmark symptom of many chronic diseases, was assessed using the Medical Research Council (MRC) dyspnea scale [41].

### Statistical Analyses

Differences in proportions between physical activity groups (inactive vs. active) were assessed using the Fisher exact test. The differences in means for count variables (eg, number of chronic conditions, number of SD behaviors) between physical activity groups were assessed using Tweedie regression. The odds of scoring higher on an ordinal scale variable was assessed using a cumulative link regression [42]. Post hoc comparisons of proportions within a given ordinal level of these models were evaluated using estimated marginal means [43]. A generalized additive model (GAM) was used to determine the effect of engaging with SD behaviors on the likelihood of performing a sufficient amount of MVPA [26]. The dependent (outcome) variable in our GAM was the binary variable indicating whether a participant’s total MVPA was 600 or more MET-minutes/week (eg, inactive vs. active). The covariates used in the GAM were selected using a gradient boosting scheme as outlined in Multimedia Appendix 1. All statistical comparisons were considered significant if *P*<.05.

## Results

### Overall Sample Characteristics

The descriptive characteristics of survey respondents are reported in Table 1. The descriptive characteristics of the entire cohort indicate that our participants were a relatively healthy, educated, and affluent sample of the general population. There was a roughly equal distribution of male and female, middle-aged respondents in both activity groups.

**Table 1:** Descriptive characteristics by physical activity group.

Characteristics				Inactive (n=1864)		Active (n=2955)		Total (N=4819)
**Demographics**								
	Age (years), median (IQR)		46 (36-59)		45 (34-59)		46 (35-59)	
	**Gender, n (%)**							
		Female	947 (50.8)		1527 (51.7)		2474 (51.3)	
		Male	917 (49.2)		1428 (48.3)		2345 (48.7)	
	BMI (kg/m^2^), median (IQR)		28.3 (24.8-33.4)		25.8 (23.1-29.8)^a^		26.7 (23.6-31.0)	
	**Physical health, median (IQR)**							
		Number of chronic health conditions	1 (0-2)		0 (0-1)^a^		1 (0-2)	
		Breathlessness (Medical Research Council score)	1 (1-2)		1 (1-1)^a^		1 (1-2)	
	**Self-reported general health, n (%)**							
		Poor	8 (0.4)		4 (0.1)^a^		12 (0.2)	
		Fair	166 (8.9)		55 (1.9)^a^		207 (4.3)	
		Good	597 (32.0)		490 (16.6)^a^		1006 (20.9)	
		Very good	882 (47.3)		1579 (53.4)^a^		2319 (48.1)	
		Excellent	211 (11.3)		827 (28.0)^a^		1275 (26.5)	
**Socioeconomic status, n (%)**								
	**Educational attainment**							
		Less than high school	5 (0.3)		8 (0.3)^a^		13 (0.3)	
		High school	91 (4.9)		75 (2.5)^a^		166 (3.4)	
		Some college no degree	320 (17.2)		289 (9.8)^a^		609 (12.6)	
		Associate degree, n (%)	168 (9.0)		245 (8.3)^a^		413 (8.6)	
		Bachelor’s degree	639 (34.3)		1072 (36.3)^a^		1711 (35.5)	
		Master’s degree	444 (23.8)		827 (28.0)^a^		1271 (26.4)	
		Doctoral/professional degree	197 (10.6)		439 (14.9)^a^		636 (13.2)	
	**Household income, n (%)**							
		Less than US $20,000	119 (6.4)		105 (3.6)^a^		224 (4.6)	
		US $20,000 to US $39,000	223 (12.0)		230 (7.8)^a^		453 (9.4)	
		US $40,000 to US $59,000	265 (14.2)		386 (13.1)^a^		651 (13.5)	
		US $60,000 to US $79,000	291 (15.6)		444 (15.0)^a^		735 (15.3)	
		US $80,000 to US $99,000	193 (10.4)		374 (12.7)		567 (11.8)	
		US $100,000 to US $149,000	325 (17.4)		632 (21.4)^a^		957 (19.9)	
		US $150,000 or more	240 (12.9)		548 (18.5)^a^		788 (16.4)	
		Prefer not to say	208 (11.2)		236 (8.0)^a^		444 (9.2)	
	**Employment status**							
		Not working, n (%)	701 (37.6)		915 (31.0)^a^		1616 (33.5)	
		Working, n (%)	1163 (62.4)		2040 (69.0)^a^		3203 (66.5)	
		Household size (number of persons), median (IQR)	2 (2-4)		2 (2-4)		2 (2-4)	

^a^Significantly different from the corresponding value (or proportion) of the inactive group, *P*<.05.

### Physical Activity

The self-reported levels of MVPA within the work, transport, and recreational domains are reported in Table 2. Unsurprisingly, respondents who were physically active reported higher amounts of recreational work and thus higher total MVPA than their inactive counterparts (*P*<.001); 61.31% (2955/4819) of our cohort were meeting the minimum WHO recommendations for weekly MVPA at the time of the survey.

**Table 2:** Physical activity and mental health during the early COVID-19 pandemic response.

Variable			Inactive (n=1864)	Active (n=2955)		Total (N=4819)	
**Physical activity**							
	**MVPA^a^ by GPAQ^b^ domain (MET^c^-minutes/week), median (IQR)**						
		Recreation	0 (0-0)	2160 (960-4080)^d^		720 (0-2760)	
		Work	0 (0-0)	0 (0-120)^d^		0 (0-0)	
		Transport	0 (0-0)	0 (0-480)^d^		0 (0-0)	
		Total	0 (0-120)	3060 (1680-5040)^d^		1320 (0-3840)	
	**Sedentary behavior**						
		Sitting time (minutes/day), median (IQR)	480 (360-660)	420 (300-600)^d^		420 (300-600)	
		At-risk sitting time (≥480 minutes/day), n (%)	1110 (59.5)	1429 (48.4)^d^		2539 (52.7)	
	**Self-monitoring behavior, n (%)**						
		Wearable device	403 (21.6)	1556 (52.7)^d^		1959 (40.7)	
**Mental health**							
	**Depression (PHQ-9^e^), median (IQR)**						
		Score	6 (3-11)		4 (2-8)^d^	5 (2-9)	
	**Symptom category, n (%)**						
		None (0-4)	748 (40.1)		1533 (51.9)^d^	2281 (47.3)	
		Mild (5-9)	565 (30.3)		873 (29.5)^d^	1438 (29.8)	
		Moderate (10-14)	277 (14.9)		328 (11.1)^d^	605 (12.6)	
		Moderately severe (15-19)	159 (8.5)		146 (4.9)^d^	305 (6.3)	
		Severe (20-27)	115 (6.2)		75 (2.5)^d^	190 (3.9)	
	**Anxiety (GAD-7^f^, median (IQR)**						
		Score	6 (2-10)		4 (1-8)^d^	5 (2-9)	
	**Symptom category, n (%)**						
		None (0-4)	820 (44.0)		1502 (50.8)^d^	2322 (48.2)	
		Mild (5-9)	500 (26.8)		848 (28.7)^d^	1348 (28.0)	
		Moderate (10-14)	303 (16.3)		356 (12.0)^d^	659 (13.7)	
		Severe (15-21)	241 (12.9)		249 (8.4)^d^	190 (3.9)	
	**Well-being (WHO-5^g^), median (IQR)**						
		Score	10 (5-16)		14 (10-18)^d^	13 (8-17)	
	**Symptom category, n (%)**						
		Okay (13-25)	727 (39.0)		1764 (59.7)^d^	2491 (51.7)	
		Poor (0-12)	1137 (61.0)		1191 (40.3)^d^	2328 (48.3)	

^a^MVPA: moderate-to-vigorous physical activity.

^b^GPAQ: Global Physical Activity Questionnaire.

^c^MET: metabolic equivalent of task.

^d^Significantly different from the corresponding value (or proportion) of the inactive group, *P*<.05.

^e^PHQ-9: 9-item Patient Health Questionnaire Scale.

^f^GAD-7: 7-item Generalized Anxiety Disorder Scale.

^g^WHO-5: 5-item World Health Organization Well-Being Index

### Social Distancing

The SD behaviors reported by the cohort are presented in Table 3. The active group was roughly 70% more likely to leave the house more frequently than their physically inactive counterparts (odds ratio [OR] 1.70, 95% CI 1.53-1.90; *P*<.001). Moreover, respondents in the active group were more likely to engage in a greater total number of SD behaviors (OR 1.10, 95% CI 1.09-1.12; *P*<.001). Specifically, physically active participants were significantly more likely (*P*<.001) to wear a face mask in public (SD1), avoid close and physical contact with others (SD2 and SD10), avoid places where people gather (SD3), work from home (SD4), limit their use of public transport (SD6), restrict their travel outside the house (SD8), and to reduce their time/number of trips to shops to obtain food and supplies (SD11). Conversely, respondents in the physically *inactive* group were more likely (*P*<.001) to avoid physical activity outside of the house (SD9).

**Table 3:** Pandemic burden, social distancing behaviors, and perceptions of fear associated with coronavirus by physical activity group during the early COVID-19 pandemic response.

Variable				Inactive (n=1864)		Active (n=2955)		Total (N=4819)
**Pandemic burden at time of survey**								
	Confirmed cases in the state (per 100,000 persons), median (IQR)		229 (168-447)		222 (166-421)		225 (167-430)	
	Recovered cases in the state (per 100,000 persons), median (IQR)		0 (0-94)		29 (0-98)^a^		0 (0-97)	
	Active cases in the state (per 100,000 persons), median (IQR)		186 (93-350)		180 (87-304)^a^		182 (88-321)	
	Deaths in the state (per 100,000 persons), median (IQR)		10.1 (7.2-23.1)		9.7 (7.3-19.4)		9.9 (7.2-20.7)	
	Duration of social distancing guidelines/restriction (weeks), median (IQR)		10.7 (10.0-14.2)		10.6 (9.8-13.8)^a^		10.6 (9.8-14.0)	
	Government stringency index, median (IQR)		73.2 (70.8-76.9)		73.2 (70.8-76.9)		73.2 (70.8-76.9)	
	Active authority-mandated lockdown/shelter-in-place/etc, n (%)		1342 (72.0)		2338 (79.1)^a^		3680 (76.4)	
**SD^b^ behaviors**								
	**Frequency of leaving the house, n (%)**							
		Less than once per week	183 (9.8)		110 (3.7)^a^		291 (6.0)	
		Once per week	274 (14.7)		278 (9.4)^a^		552 (11.5)	
		A few times per week	634 (34.0)		926 (31.3)^a^		1560 (32.4)	
		Once per day	436 (23.4)		1042 (35.3)^a^		1478 (30.7)	
		Multiple times per day	337 (18.1)		599 (20.3)^a^		936 (19.4)	
	SD1: Wearing a face mask in public, n (%)		1371 (73.6)		2542 (86.0)^a^		3913 (81.2)	
	SD2: Avoid close contact with others in social circle, n (%)		1420 (76.2)		2605 (88.2)^a^		4025 (83.5)	
	SD3: Avoid places where many people gather, n (%)		1478 (79.3)		2702 (91.4)^a^		4180 (86.7)	
	SD4: Working from home, n (%)		970 (52.0)		1779 (60.2)^a^		2749 (57.0)	
	SD5: Limiting time spent outside of house, n (%)		1112 (59.7)		1840 (62.3)		2952 (61.3)	
	SD6: Limiting the use of public transport, n (%)		1377 (73.9)		2546 (86.2)^a^		3923 (81.4)	
	SD7: Currently undergoing self-isolation/quarantine, n (%)		1015 (54.5)		1581 (53.5)		2596 (53.9)	
	SD8: Restricting travel outside of the house, n (%)		1233 (66.1)		2175 (73.6)^a^		3408 (70.7)	
	SD9: Avoiding physical activity outside of the house, n (%)		664 (35.6)		546 (18.5)^a^		1210 (25.1)	
	SD10: Avoid physical contact with others, n (%)		1511 (81.1)		2789 (94.4)^a^		4300 (89.2)	
	SD11: Reducing time/number of trips to shops for food/supplies/etc, n (%)		1410 (75.6)		2631 (89.0)^a^		4041 (83.9)	
	Total number of SD behaviors (sum SD1-11), median (IQR)		9 (6-10)		9 (7-10)^a^		9 (7-10)	
**Perceived fears of COVID-19^c^, median (IQR)**								
	Afraid of being infected with COVID-19		5 (4-6)		5 (4-6)		5 (4-6)	
	Afraid of unknowingly spreading COVID-19		6 (5-7)		6 (5-7)		6 (5-7)	

^a^Significantly different from corresponding value (or proportion) of the inactive group, *P*<.05.

^b^SD: social distancing.

^c^Likert-type item (1=strongly disagree; 7=strongly agree).

### Mental Health

The prevailing mental health of participants in the sampled cohort is reported in Table 2. Raw scores for depression and anxiety were lower for the physically active compared with the inactive group (*P*<.001). In support of these observations, respondents in the physically active group displayed lower odds of reporting more severe symptoms of depression (OR 0.72, 95% CI 0.69-0.74; *P*<.001) and anxiety (OR 0.81, 95% CI 0.76-0.86; *P*<.001). The raw score for mental well-being was overall higher for participants in the active compared with the inactive group (*P*<.001). There were also lower odds of the respondents’ well-being score falling below 13 (ie, “poor well-being”) [30] for those participants in the physically active group (OR 0.43, 95% CI 0.38-0.49; *P*<.001).

### Pandemic Burden and Fear

Indicators of the burden of the pandemic and fears associated with COVID-19 are reported in Table 3. Overall, SD rules/guidelines had been active for approximately 2-3 months at the time of survey for the entire cohort—this duration was slightly lower in the physically active group (*P*=.003). The number of recovered COVID-19 cases (per 100,000 persons) was higher (*P*=.002), whereas the number of active cases was marginally lower (*P*=.005) in the active compared with the inactive group. The burden of deaths due to COVID-19 was similar between physical activity groups. There was a marginally higher proportion of respondents under an authority-mandated “lockdown” at the time of the survey in the active group (*P*<.001). The perceived fear of becoming infected with COVID-19 and the fear associated with unknowingly spreading the virus were similar between groups.

### Sedentary and Self-Monitoring Behavior

Sedentary behavior (minutes/day) was slightly lower in the active group (*P*<.001; Table 3). In addition, the proportion of participants who reported that time spent sitting/reclining exceeded 8 hours per day was marginally lower in the physically active group (*P*<.001; Table 3). There was a greater proportion of respondents using a wearable device to track their physical activity in the active compared with the inactive group (*P*<.001; Table 3).

### Socioeconomic Status and Physical Health

The cohort indicators of socioeconomic status and physical health are reported in Table 1. There was a greater proportion of respondents who were employed at the time of the survey in the active group (*P*<.001). Moreover, there were higher (*P*<.001) odds of possessing a higher level of educational attainment (OR 1.58, 95% CI 1.42-1.75) and household income (OR 1.16, 95% CI 1.09-1.23) for the active group. BMI, the number of chronic conditions, and the experience of breathlessness (MRC score) during daily activities were slightly higher in the inactive compared with the physically active group (*P*<.001). Lastly, there were greater odds of self-reporting better general health (OR 3.15, 95% CI 2.81-3.53; *P*<.001).

### Logistic Generalized Additive Modeling

**Figure 1:**
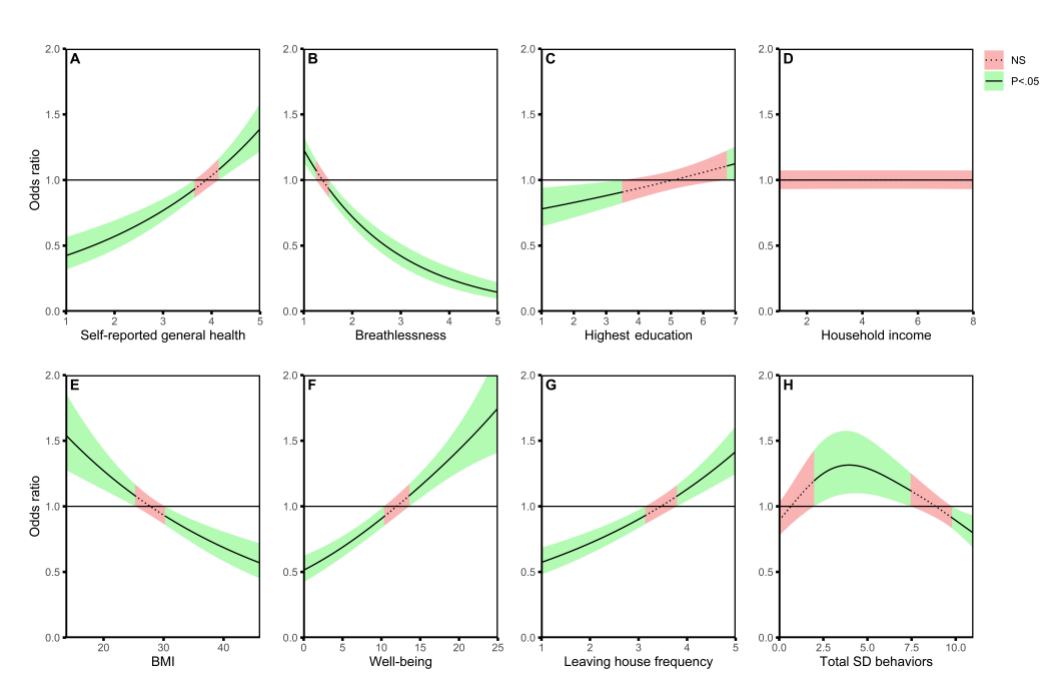
Nonlinear effects of physical and mental health, and social distancing vigilance on the odds of meeting WHO recommendations for physical activity during the early COVID-19 pandemic response. The solid lines indicate the nonlinear trend in the odds of meeting WHO recommendations for physical activity for the corresponding covariate. The green-shaded regions denote the range of values of a covariate where the odds ratio for meeting WHO recommendations for physical activity is significantly different (*P*<.05) from 1.00 (ie, equivocal odds). Conversely, the red-shaded regions indicate the values of the respective covariate where the odds ratio is not different from 1.00. Note that self-reported general health (panel A), highest level of educational attainment (panel B), and household income (panel D) were input into the generalized additive model as ordinal variables. As such, the integer values correspond to the ordinal categorical levels of each covariate in order of lowest to highest category (eg, self-reported general health: 1=very poor; 5=excellent). NS: not significant; SD: social distancing.

Tables 4 and 5 show the results of the logistic GAM parametric and smooth terms, respectively, used to determine the likelihood of engaging in higher amounts of physical activity. The coefficient of determination, *R*^2^, for the logistic GAM was 0.37 and there was a significant improvement over an intercept-only (null) model (*P*<.001) [44]. The variance of the random effect of US state was not significant (σ^2^=1.16 × 10^–13^; 95% CI –5.47 × 10^–7^ to 8.92 × 10^–7^; *P*=.62). The use of a wearable device to track physical activity (ie, self-monitoring behavior), wearing a facemask in public (SD1), limiting the use of public transport and the number of trips to the shops (SD6 and SD11), and avoiding close physical contact with others were all positively associated with greater odds of performing sufficient (≥600 MET-minutes/week) amounts of MVPA during the early COVID-19 pandemic (*P*<.005). Avoiding physical activity outside the house was negatively associated with the odds of being physically active (*P*<.002). The nonlinear trends in ORs for all smooth terms are illustrated in Figure 1. The odds of being physically active (total MVPA ≥600 MET-minutes/week) tended to rise with greater self-reported general health (Figure 1A), higher levels of educational attainment (Figure 1C), increasing mental well-being (Figure 1F), and higher frequencies of leaving the house (Figure 1G). Conversely, the odds of being sufficiently active during the early pandemic decreased with increasing breathlessness (Figure 1B) and BMI (Figure 1E). Importantly, the effect of engaging with a higher number of SD behaviors on the odds of performing sufficient MVPA during the pandemic was seemingly biphasic (Figure 1H). For example, participating in 3-8 total SD behaviors was coupled with greater odds, whereas engaging with 10 or more SD behaviors was associated with lower odds of performing sufficient MVPA.

**Table 4:** Factors influencing physical activity level during the COVID-19 pandemic as determined by logistic generalized additive modeling (N=4819).^a^

Parametric terms	Statistics^b^		
	OR	95% CI	*P* value
Intercept	*0.24*	*0.19-0.32*	*<.001*
“At-risk” sedentary behavior (reference = less than 8 hours/day)	*0.71*	*0.62-0.83*	*<.001*
Wearable device	*3.27*	*2.82-3.79*	*<.001*
SD1: Wearing a facemask in public	*1.43*	*1.14-1.79*	*.003*
SD2: Avoiding close contact with others	1.20	0.91-1.59	.199
SD3: Avoiding places where many people gather	1.26	0.91-1.73	.193
SD6: Limiting the use of public transport	*1.47*	*1.19-1.83*	*.001*
SD9: Avoiding physical activity outside the house	*0.52*	*0.43-0.63*	*<.001*
SD10: Avoiding physical contact with others	*1.79*	*1.26-2.56*	*.002*
SD11: Reducing time/number of trips to shops for supplies	*1.56*	*1.19-2.05*	*.002*

^a^Parameter estimates for the parametric (linear) terms in the model are reported as the exponentiated log-odds ratio (ie, OR) and corresponding 95% CI. The OR indicates the odds of meeting the World Health Organization’s minimum physical activity recommendations (≥600 MET-minutes/week) per unit change in the respective covariate. The Benjamini–Hochberg method was used to adjust *P* values to reduce the false discovery rate.

^b^Significant values are in italic.

**Table 5:** Smooth terms influencing physical activity level during the COVID-19 pandemic as determined by logistic generalized additive modeling (N=4819).^a^

Smooth terms	Statistics^b^	
	*edf*	*P* value
BMI	*0.85*	*<.001*
Highest level of educational attainment	*0.65*	*.001*
Household income	*0.01*	*.023*
Number of chronic health conditions	0.00	.197
Self-reported general health	*0.90*	*<.001*
Breathlessness	*0.96*	*<.001*
Frequency of leaving the house	*0.91*	*<.001*
Total number of SD behaviors	*1.23*	*<.001*
Well-being	*1.19*	*<.001*

^a^The smooth terms included in the generalized additive model are summarized by their estimated degrees of freedom (*edf*). The Benjamini–Hochberg method was used to adjust *P* values to reduce the false discovery rate.

^b^Significant values are in italic.

## Discussion

### Principal Findings

The principal findings of this study were threefold: (1) physically active respondents were more likely to engage in SD behaviors; (2) the influence of engaging with SD behaviors on physical activity during the early pandemic was nonlinear; and (3) higher scores for mental well-being were a positive mediator of physical activity participation. These findings highlight the complex nature by which SD vigilance and mental health have impacted on the physical activity habits of the general population during the early COVID-19 pandemic.

### Did Social Distancing Affect Physical Activity During the Early Pandemic Response?

We originally hypothesized that engaging in more SD behaviors would increase the likelihood of being *physically inactive* during the early pandemic response. Our findings only partly confirm this hypothesis. For example, participants who minimized their public exposure by leaving the house less than “once per day” were less likely to be physically active (Figure 1G). Furthermore, respondents were less likely to meet the minimum WHO recommendations for weekly MVPA if they reported that they were “avoiding physical activity outside the house” (SD9) at the time of the survey (Tables 4 and 5). However, the relationship between the total number of SD behaviors and physical activity was much less straightforward (Figure 1H). Certainly, individuals engaging with 10 or more of the surveilled SD behaviors (highly vigilant) were at lower odds of being physically active during the pandemic. Interestingly, however, it appeared that if a participant engaged with a moderate number of SD behaviors (3-7 total), they were at higher odds of meeting the minimum WHO recommendations for weekly MVPA. This nonlinear relationship between SD vigilance and physical activity is novel, insofar as it describes a potential “tipping point” phenomenon: too much is bad, yet a moderate amount is good. But which of the SD behaviors are specifically associated with being physically active?

The cross-sectional analyses of SD behaviors within our cohort (Table 3) appear to suggest that those individuals who were *physically active* during the early pandemic were more frequently wearing a facemask in public (SD1), avoiding close and physical contact with others (SD2 and SD10), avoiding places where people gather (SD3), working from home (SD4), and more often limiting their public exposure by restricting their use of public transport and travel outside the house (SD6, SD8, and SD11). These observations are complemented by the logistic GAM analysis (Tables 4 and 5), whereby SD1, SD6, SD10, and SD11 were all associated with significantly higher odds of meeting the minimum WHO requirements for weekly MVPA at the time of the survey. The following question arises: why does engaging in *some* but not *all* SD measures appear to be positively associated with physical activity? It is difficult to offer any substantive explanation for these observations given the data at hand. Notwithstanding this point, it is known that physical activity level is positively associated with health literacy [45-47]. Thus, it is at least conceivable that participants who regularly engaged in more physical activity may have been better informed and aware of public health initiatives and were thus more likely to follow SD guidelines. The opposite is also plausible: those respondents who engaged with a moderate number of SD behaviors may also be more likely to heed other public health advice, such as recommendations for physical activity. However, this positive effect is only apparent up until the individual engages in nearly all (≥10) of the surveilled SD behaviors, after which it is likely that simultaneously engaging in these behaviors becomes prohibitive to accumulating sufficient weekly MVPA. It will be of great interest to assess whether vigilance with SD behaviors remains nonlinearly associated with physical activity level at our planned follow-up survey rounds.

### Did Mental Health Affect Physical Activity During The Early Pandemic Response?

It is becoming clear that extended periods of social isolation, as imposed by public health measures, have negatively impacted on mental health during the COVID-19 pandemic [18-21]. This point is particularly concerning given that mental health may affect physical activity, and vice versa [22,23]. Indeed, cross-sectional analysis of our cohort tended to corroborate the above findings, whereby respondents in the *physically active* group reported higher well-being scores, and less symptoms of depression and anxiety compared with those in the inactive group (Table 2). However, among the 3 indicators of mental health, it was only the raw score for mental well-being (ie, WHO-5) that was selected as a covariate in the boosted GAM model (see Multimedia Appendix 1 for details). Specifically, we observed that raw scores for mental well-being greater than 13 were associated with meeting the WHO recommendations for weekly MVPA. However, participants with raw WHO-5 scores below this value (ie, “poor well-being”) [30] were more likely to be *physically inactive* during the early pandemic response. Overall, the above findings support our secondary hypothesis that poorer mental health was associated with less physical activity during the early pandemic response.

### What Other Factors Influenced Physical Activity in Our Cohort?

Respondents were more likely to be *physically active* if they were sedentary for less than 8 hours per day (Tables 4 and 5). This observation is perhaps not surprising given that daily hours are finite, and less time spent engaging with one behavior (ie, sitting) affords more time for another behavior (ie, physical activity) [48]. Those participants who reported that they used a wearable device to monitor their own physical activity were also more likely to accumulate sufficient weekly MVPA during the early pandemic response (Tables 4 and 5). This finding is consistent with the idea that objective self-monitoring, using wearable technologies, is a behavior change tool that is effective in reducing sedentary time and increasing physical activity in adults [49,50].

### Methodological Considerations

Many investigators have argued that SD policies for minimizing spread of COVID-19 may worsen an existing global health crisis, that is, the physical inactivity pandemic [13,51]. Emerging research has vindicated these concerns by illustrating that physical activity of the public has declined during the COVID-19 pandemic [5-10]. Given that extending the recall period of the GPAQ to far beyond the past 7 days is likely to confound data with recall bias [52], we have not reported MVPA of our participants from a time before the pandemic began. As such, our data do not allow us to comment on whether physical activity truly declined during the early pandemic period in our cohort. For similar reasons, we are unable to directly comment on whether mental health status, as assessed via the GAD-7, WHO-5, and PHQ-9, worsened during the early pandemic in our cohort. A further consideration is that while our cohort was large, it is unlikely that our sample is representative of the greater US population. Our cohort was a convenience sample recruited via social media, a method of sampling known to recruit greater proportions of adults with higher levels of educational attainment than the general population [53]. Indeed, our cohort was a highly educated and affluent sample of the general population. We therefore emphasize that our findings may not apply to a more representative sample of a larger US population with greater socioeconomic diversity than that observed in this study.

### Implications of Our Findings

Given that SD has arguably encouraged a public shift toward sedentariness, it is essential that we identify those factors of a person’s “pandemic experience” which have contributed to this decline in physical activity. Our findings offer 4 major insights into the potential mediators of physical activity during the early pandemic response. First, we report that individuals with poor mental well-being were likely to be *physically inactive* during the early pandemic. Second, our data provide strong evidence that “getting outside” the house encourages sufficient weekly MVPA, notwithstanding any SD guidelines/restrictions that may be active at the time. Third, individuals demonstrating self-monitoring behavior via wearable activity trackers were more likely to accumulate sufficient weekly MVPA. Lastly, the extent to which SD vigilance impacts on physical activity is complex, insofar as engaging in a moderate number of SD behaviors (3-7 total) was associated with being physically active, while engaging in too many SD behaviors (≥10 total) was seemingly detrimental to engaging in adequate amounts of physical activity. This last observation may be telling of the challenges faced by the public when regulating their own vigilance with SD behaviors. We speculate that this finding may be a symptom of the belief that either (1) adhering to all SD behaviors takes priority over all other health promoting behaviors during the pandemic or (2) one cannot safely perform SD while being physically active, particularly outside the house.

In light of these findings, we suggest that public health messaging of SD guidelines may be improved to promote physical activity during the pandemic by including specific advice outlining how to be physically active “outdoors” in a COVID-safe manner (eg, targeted infographics) [54,55], and by clearly portraying the benefits of regular exercise on mental health [56-58]. In such messaging, it would be worth mentioning that evidence suggests being physically fit confers a degree of immunity protection [59], and may reduce morbid outcomes associated with COVID-19, such as hospitalizations [60,61]. Lastly, our data indicate there may be value in specifically encouraging the use of wearable devices to self-monitor physical activity levels.

### Conclusions

The recent availability of COVID-19 vaccines has marked the beginning of our recovery from this global pandemic [62]. However, until vaccination rates approach levels that confer “herd immunity” against the virus, SD measures will remain part of our COVID-normal existence for the foreseeable future. If we fail to recognize the impact that SD bears on physical activity, we may yet observe a “final wave” of chronic lifestyle diseases once the pandemic recedes. The findings of our investigation support the viewpoint that physical activity promotion should be more heavily integrated into the public health messaging of physical/SD guidelines during this current pandemic, and that any of these may precipitate in the future.

## Appendix

Multimedia Appendix 1 Methodological approach.

## Abbreviations

GAD-7: 7-item Generalized Anxiety Disorder Scale
GAM: generalized additive model
GPAQ: Global Physical Activity Questionnaire
MET: metabolic equivalent of task
MRC: Medical Research Council
MVPA: moderate-to-vigorous physical activity
OR: odds ratio
PAWS: Physical Activity and Well-being Survey
PHQ-9: 9-item Patient Health Questionnaire
SD: social distancing
WHO: World Health Organization

## References

[1] Centers for Disease Control and Prevention (2020). Social Distancing - Keep a Safe Distance to Slow the Spread.

[2] Honein MA, Christie A, Rose DA, Brooks JT, Meaney-Delman D, Cohn A, Sauber-Schatz EK, Walker A, McDonald LC, Liburd LC, Hall JE, Fry AM, Hall AJ, Gupta N, Kuhnert WL, Yoon PW, Gundlapalli AV, Beach MJ, Walke HT, CDC COVID-19 Response Team (2020). Summary of Guidance for Public Health Strategies to Address High Levels of Community Transmission of SARS-CoV-2 and Related Deaths, December 2020. MMWR Morb Mortal Wkly Rep.

[3] World Health Organiziation Coronavirus Disease (COVID-19) Advice for the Public.

[4] UK Government Guidance: Coronavirus (COVID-19): Meeting With Others Safely (Social Distancing).

[5] Lesser IA, Nienhuis CP (2020). The Impact of COVID-19 on Physical Activity Behavior and Well-Being of Canadians. Int J Environ Res Public Health.

[6] Meyer J, McDowell C, Lansing J, Brower C, Smith L, Tully M, Herring M (2020). Changes in Physical Activity and Sedentary Behavior in Response to COVID-19 and Their Associations with Mental Health in 3052 US Adults. Int J Environ Res Public Health.

[7] Tison GH, Avram R, Kuhar P, Abreau S, Marcus GM, Pletcher MJ, Olgin JE (2020). Worldwide Effect of COVID-19 on Physical Activity: A Descriptive Study. Ann Intern Med.

[8] Qin F, Song Y, Nassis GP, Zhao L, Dong Y, Zhao C, Feng Y, Zhao J (2020). Physical Activity, Screen Time, and Emotional Well-Being during the 2019 Novel Coronavirus Outbreak in China. Int J Environ Res Public Health.

[9] Wilke J, Mohr L, Tenforde AS, Edouard P, Fossati C, González-Gross Marcela, Sánchez Ramírez Celso, Laiño Fernando, Tan B, Pillay JD, Pigozzi F, Jimenez-Pavon D, Novak B, Jaunig J, Zhang M, van Poppel M, Heidt C, Willwacher S, Yuki G, Lieberman DE, Vogt L, Verhagen E, Hespanhol L, Hollander K (2021). A Pandemic within the Pandemic? Physical Activity Levels Substantially Decreased in Countries Affected by COVID-19. Int J Environ Res Public Health.

[10] Caputo EL, Reichert FF (2020). Studies of Physical Activity and COVID-19 During the Pandemic: A Scoping Review. J Phys Act Health.

[11] Lavie CJ, Ozemek C, Carbone S, Katzmarzyk PT, Blair SN (2019). Sedentary Behavior, Exercise, and Cardiovascular Health. Circ Res.

[12] Stamatakis E, Gale J, Bauman A, Ekelund U, Hamer M, Ding D (2019). Sitting Time, Physical Activity, and Risk of Mortality in Adults. J Am Coll Cardiol.

[13] Kohl HW, Craig CL, Lambert EV, Inoue S, Alkandari JR, Leetongin G, Kahlmeier S, Lancet PASWG (2012). The pandemic of physical inactivity: global action for public health. Lancet.

[14] World Health Organization (2009). Global Health Risks: Mortality and Burden of Disease Attributable to Selected Major Risks.

[15] Jiménez-Pavón D, Carbonell-Baeza A, Lavie CJ (2020). Physical exercise as therapy to fight against the mental and physical consequences of COVID-19 quarantine: Special focus in older people. Prog Cardiovasc Dis.

[16] Banerjee D, Rai M (2020). Social isolation in Covid-19: The impact of loneliness. Int J Soc Psychiatry.

[17] Killgore WDS, Cloonan SA, Taylor EC, Dailey NS (2020). Loneliness: A signature mental health concern in the era of COVID-19. Psychiatry Res.

[18] Vindegaard N, Benros ME (2020). COVID-19 pandemic and mental health consequences: Systematic review of the current evidence. Brain Behav Immun.

[19] Pierce M, Hope H, Ford T, Hatch S, Hotopf M, John A, Kontopantelis E, Webb R, Wessely S, McManus S, Abel KM (2020). Mental health before and during the COVID-19 pandemic: a longitudinal probability sample survey of the UK population. The Lancet Psychiatry.

[20] Xiong J, Lipsitz O, Nasri F, Lui LMW, Gill H, Phan L, Chen-Li D, Iacobucci M, Ho R, Majeed A, McIntyre RS (2020). Impact of COVID-19 pandemic on mental health in the general population: A systematic review. J Affect Disord.

[21] Dubey S, Biswas P, Ghosh R, Chatterjee S, Dubey MJ, Chatterjee S, Lahiri D, Lavie CJ (2020). Psychosocial impact of COVID-19. Diabetes Metab Syndr.

[22] Steinmo S, Hagger-Johnson G, Shahab L (2014). Bidirectional association between mental health and physical activity in older adults: Whitehall II prospective cohort study. Prev Med.

[23] Azevedo Da Silva M, Singh-Manoux A, Brunner EJ, Kaffashian S, Shipley MJ, Kivimäki Mika, Nabi H (2012). Bidirectional association between physical activity and symptoms of anxiety and depression: the Whitehall II study. Eur J Epidemiol.

[24] Armstrong T, Bull F (2006). Development of the World Health Organization Global Physical Activity Questionnaire (GPAQ). J Public Health.

[25] World Health Organization (2005). WHO STEPS Surveillance Manual: The WHO STEPwise Approach to Chronic Disease Risk Factor Surveillance.

[26] Bull FC, Al-Ansari SS, Biddle S, Borodulin K, Buman MP, Cardon G, Carty C, Chaput J, Chastin S, Chou R, Dempsey PC, DiPietro L, Ekelund U, Firth J, Friedenreich CM, Garcia L, Gichu M, Jago R, Katzmarzyk PT, Lambert E, Leitzmann M, Milton K, Ortega FB, Ranasinghe C, Stamatakis E, Tiedemann A, Troiano RP, van der Ploeg HP, Wari V, Willumsen JF (2020). World Health Organization 2020 guidelines on physical activity and sedentary behaviour. Br J Sports Med.

[27] Kroenke K, Spitzer RL, Williams JB (2001). The PHQ-9: validity of a brief depression severity measure. J Gen Intern Med.

[28] Spitzer RL, Kroenke K, Williams JBW, Löwe Bernd (2006). A brief measure for assessing generalized anxiety disorder: the GAD-7. Arch Intern Med.

[29] Topp CW, Østergaard Søren Dinesen, Søndergaard Susan, Bech P (2015). The WHO-5 Well-Being Index: a systematic review of the literature. Psychother Psychosom.

[30] World Health Organization (1998). Wellbeing Measures in Primary Health Care - The DepCare Project.

[31] Guidotti E, Ardia D (2020). COVID-19 Data Hub. JOSS.

[32] Hale T, Angrist N, Goldszmidt R, Kira B, Petherick A, Phillips T, Webster S, Cameron-Blake E, Hallas L, Majumdar S, Tatlow H (2021). A global panel database of pandemic policies (Oxford COVID-19 Government Response Tracker). Nat Hum Behav.

[33] Ekelund U, Brown WJ, Steene-Johannessen J, Fagerland MW, Owen N, Powell KE, Bauman AE, Lee I (2019). Do the associations of sedentary behaviour with cardiovascular disease mortality and cancer mortality differ by physical activity level? A systematic review and harmonised meta-analysis of data from 850 060 participants. Br J Sports Med.

[34] Eime RM, Charity MJ, Harvey JT, Payne WR (2015). Participation in sport and physical activity: associations with socio-economic status and geographical remoteness. BMC Public Health.

[35] Gidlow C, Johnston LH, Crone D, Ellis N, James D (2016). A systematic review of the relationship between socio-economic position and physical activity. Health Education Journal.

[36] Martínez-González M A, Martínez J A, Hu F, Gibney M, Kearney J (1999). Physical inactivity, sedentary lifestyle and obesity in the European Union. Int J Obes Relat Metab Disord.

[37] Rütten A, Abel T, Kannas L, von Lengerke T, Lüschen G, Diaz JA, Vinck J, van der Zee J (2001). Self reported physical activity, public health, and perceived environment: results from a comparative European study. J Epidemiol Community Health.

[38] Opdal IM, Larsen LS, Hopstock LA, Schirmer H, Lorem GF (2020). A prospective study on the effect of self-reported health and leisure time physical activity on mortality among an ageing population: results from the Tromsø study. BMC Public Health.

[39] Booth FW, Roberts CK, Thyfault JP, Ruegsegger GN, Toedebusch RG (2017). Role of Inactivity in Chronic Diseases: Evolutionary Insight and Pathophysiological Mechanisms. Physiol Rev.

[40] Booth FW, Roberts CK, Laye MJ (2012). Lack of exercise is a major cause of chronic diseases. Compr Physiol.

[41] Fletcher Cm, Elmes Pc, Fairbairn As, Wood Ch (1959). The significance of respiratory symptoms and the diagnosis of chronic bronchitis in a working population. Br Med J Aug 29.

[42] Christensen R (2019). ordinal—Regression Models for Ordinal Data.

[43] Lenth R (2021). emmeans—Estimated Marginal Means, aka Least-Squares Means.

[44] Nagelkerke NJD (1991). A note on a general definition of the coefficient of determination. Biometrika.

[45] Cajita M, Denhaerynck K, Dobbels F, Berben L, Russell C, De Geest S (2016). Adequate Health Literacy Is Associated with Sufficient Physical Activity: Findings from the BRIGHT Study. The Journal of Heart and Lung Transplantation.

[46] Valatkaitytė V, Česnaitienė VJ (2019). Relationship Between Health Literacy, Physical Activity, Motivation and Barriers of People Aged 30–50 Years. BJSHS.

[47] Buja A, Rabensteiner A, Sperotto M, Grotto G, Bertoncello C, Cocchio S, Baldovin T, Contu P, Lorini C, Baldo V (2020). Health Literacy and Physical Activity: A Systematic Review. J Phys Act Health.

[48] Mekary RA, Willett WC, Hu FB, Ding EL (2009). Isotemporal substitution paradigm for physical activity epidemiology and weight change. Am J Epidemiol.

[49] Compernolle S, DeSmet A, Poppe L, Crombez G, De Bourdeaudhuij I, Cardon G, van der Ploeg HP, Van Dyck D (2019). Effectiveness of interventions using self-monitoring to reduce sedentary behavior in adults: a systematic review and meta-analysis. Int J Behav Nutr Phys Act.

[50] Laranjo L, Ding D, Heleno B, Kocaballi B, Quiroz JC, Tong HL, Chahwan B, Neves AL, Gabarron E, Dao KP, Rodrigues D, Neves GC, Antunes ML, Coiera E, Bates DW (2021). Do smartphone applications and activity trackers increase physical activity in adults? Systematic review, meta-analysis and metaregression. Br J Sports Med.

[51] Hall G, Laddu DR, Phillips SA, Lavie CJ, Arena R (2021). A tale of two pandemics: How will COVID-19 and global trends in physical inactivity and sedentary behavior affect one another?. Prog Cardiovasc Dis.

[52] Cross TJ, Isautier JMJ, Stamatakis E, Morris SJ, Johnson BD, Wheatley-Guy C, Taylor BJ (2021). Self-reported physical activity before a COVID-19 'lockdown': is it just a matter of opinion?. BMJ Open Sport Exerc Med.

[53] Thornton L, Batterham PJ, Fassnacht DB, Kay-Lambkin F, Calear AL, Hunt S (2016). Recruiting for health, medical or psychosocial research using Facebook: Systematic review. Internet Interv.

[54] Wedig IJ, Duelge TA, Elmer SJ (2021). Infographic. Stay physically active during COVID-19 with exercise as medicine. Br J Sports Med.

[55] Fitzpatrick J, Castricum A, Seward H, Tulloh L, Dawson E (2020). Infographic. COFIT-19: let's get moving through the COVID-19 pandemic!. Br J Sports Med.

[56] Petruzzello SJ, Landers DM, Hatfield BD, Kubitz KA, Salazar W (1991). A meta-analysis on the anxiety-reducing effects of acute and chronic exercise. Outcomes and mechanisms. Sports Med.

[57] DeBoer LB, Powers MB, Utschig AC, Otto MW, Smits JAJ (2012). Exploring exercise as an avenue for the treatment of anxiety disorders. Expert Rev Neurother.

[58] Chekroud S, Gueorguieva R, Zheutlin A, Paulus M, Krumholz H, Krystal J, Chekroud Am (2018). Association between physical exercise and mental health in 1·2 million individuals in the USA between 2011 and 2015: a cross-sectional study. The Lancet Psychiatry.

[59] Laddu DR, Lavie CJ, Phillips SA, Arena R (2021). Physical activity for immunity protection: Inoculating populations with healthy living medicine in preparation for the next pandemic. Prog Cardiovasc Dis.

[60] Brawner CA, Ehrman JK, Bole S, Kerrigan DJ, Parikh SS, Lewis BK, Gindi RM, Keteyian C, Abdul-Nour K, Keteyian SJ (2021). Inverse Relationship of Maximal Exercise Capacity to Hospitalization Secondary to Coronavirus Disease 2019. Mayo Clin Proc.

[61] Lavie CJ, Sanchis-Gomar F, Arena R (2021). Fit Is It in COVID-19, Future Pandemics, and Overall Healthy Living. Mayo Clin Proc.

[62] Centers for Disease Control and Prevention - National Center for Health Statistics (2021). Trends in Number of COVID-19 Cases and Deaths in the US Reported to CDC.

