# Supplementary material for: The Influence of Social Distancing Behaviors and Psychosocial Factors on Physical Activity During the COVID-19 Pandemic: Cross-sectional Survey Study
Source: JMIR Public Health Surveill. 2021 Sep 24;7(9):e31278. doi: 10.2196/31278 (PMC8477911; doi:10.2196/31278)
Supplement: Multimedia Appendix 1 [file publichealth_v7i9e31278_app1.docx]

**Online Appendix**

**Methods**

*Statistical analyses*

*Logistic generalized additive modeling*

A generalized additive model (GAM) was used to determine the effect of engaging with social distancing behaviors on the likelihood of performing a sufficient amount of MVPA [1]. In our study, we fit a logistic GAM to physical activity level using the binomial family from the *mgcv* R-package [2]. The output of a logistic GAM for parametric (linear) terms is directly comparable to the log-odds obtained from logistic regressions. However, any smooth terms which are fit to data cannot be characterized by a single parameter value and, instead, represent a continuous function of log-odds across the domain of observations. In essence, the logistic GAM allows for nonlinear effects of continuous and ordinal covariates on the log-odds of performing sufficient MVPA. The dependent (outcome) variable in our GAM was the binary variable indicating whether a participant’s total MVPA was ≥600 MET-min∙week^-1^ (e.g., inactive vs active).

There were 45 candidate variables available to include in the logistic GAM, inclusive of all exposures and covariates. Although the ratio of observations to covariates was large in our dataset (~108 observations per variable), it was evident during preliminary analysis that these data suffered from multicollinearity and concurvity. To address this issue, we first removed binary covariates with low (rare) observation counts (<5%). Secondly, we performed variable selection using a “probing” approach outlined by Thomas *et al.* [3]. This method of variable selection begins with augmenting the original dataset with randomly shuffled copies of the original variables. These new, shuffled data are referred to as “shadow” variables, in that they preserve the marginal distributions of the original variables, yet are uncorrelated with the target outcome variable. Gradient boosting of the GAM is then performed [4]. GAM boosting is an iterative machine learning approach that involves the repeated training of *weak learners* (i.e., single covariates) on the residuals output from the previous learner. Variable selection (via this method) is achieved by selecting the single covariate which most greatly reduces the residual scores from the previous boosting iteration [3, 4]. Gradient boosting is continued until either a maximum number of iterations is reached, or some termination criteria is achieved. According to the “probing” approach described by Thomas *et al.* [3], the boosting iterations are stopped when the first of any “shadow” variable is selected. After this point, any other variables which are selected by further boosting iterations are likely correlated with the outcome variable by chance. The “probing” approach to GAM boosting led to the selection of 18 covariates. As suggested by Simon Woods [5], a final (non-boosted) GAM was fit to the original data using these 18 covariates using the double penalty for automatic smoothness selection via the Restricted Maximal Likelihood (REML) method [6]. The parameter *γ* set to log(*N*)/2 to reduce overfitting, and an extra penalty was added to each smooth term so that it could be penalized to zero [7, 8]. US State was included as a random effect to account for the clustering of responses from participants within the same governmental region. Penalized splines were used as the basis functions for all smooth terms.

The significance of all parametric and smooth terms in the final logistic GAM were adjusted using the Benjamani-Hochberg procedure which minimized false-discovery-rate among the multiple comparisons [9]. The log-odds of parametric terms, and those predicted by smooth terms, were exponentiated to produce odds ratios (OR). However, the effect of a nonlinear smooth term on the dependent variable (i.e., odds of being *physically active*) cannot be characterized by any single number and, instead, one must investigate the nonlinear effect across the domain of the curve. Therefore, it was necessary to examine any significant (*P*<.05) smooth terms to determine which values of the covariate were odds ratios different from a reference of 1.00 (i.e., equivocal odds/no effect). Only those values of the covariate where the 95% confidence intervals of the estimated smooth did not include 1.00 were considered significant at the *α*-level of .05.

**References**

1. Bull FC, Al-Ansari SS, Biddle S, Borodulin K, Buman MP, Cardon G, et al. World Health Organization 2020 guidelines on physical activity and sedentary behaviour. Br J Sports Med. 2020;54(24):1451-62. doi: 10.1136/bjsports-2020-102955.

2. Wood SN. *mcgv*—Mixed GAM Computation Vehicle with Automatic Smoothness Estimation. 2021; Available from: <https://CRAN.R-project.org/package=mgcv>.

3. Thomas J, Hepp T, Mayr A, Bischl B. Probing for Sparse and Fast Variable Selection with Model-Based Boosting. Computational and Mathematical Methods in Medicine. 2017 2017/07/31;2017:1421409. doi: 10.1155/2017/1421409.

4. Bühlmann P, Hothorn T. Boosting Algorithms: Regularization, Prediction and Model Fitting. Statistical Science. 2007;22(4):477-505, 29.

5. Wood SN. Inference and computation with generalized additive models and their extensions. TEST. 2020 2020/06/01;29(2):307-39. doi: 10.1007/s11749-020-00711-5.

6. Wood SN. Generalized additive models: An introduction with R: CRC Press; 2017.

7. Reiss PT, Todd Ogden R. Smoothing parameter selection for a class of semiparametric linear models. Journal of the Royal Statistical Society: Series B (Statistical Methodology). 2009;71(2):505-23. doi: 10.1111/j.1467-9868.2008.00695.x.

8. Wood SN. Fast stable direct fitting and smoothness selection for generalized additive models. Journal of the Royal Statistical Society: Series B (Statistical Methodology). 2008;70(3):495-518. doi: 10.1111/j.1467-9868.2007.00646.x.

9. Benjamini Y, Hochberg Y. Controlling the False Discovery Rate: A Practical and Powerful Approach to Multiple Testing. Journal of the Royal Statistical Society: Series B (Methodological). 1995;57(1):289-300. doi: <https://doi.org/10.1111/j.2517-6161.1995.tb02031.x>.
